# Low incidence of inbreeding in a long-lived primate population isolated for 75 years

**DOI:** 10.1007/s00265-016-2236-6

**Published:** 2016-12-09

**Authors:** Anja Widdig, Laura Muniz, Mirjam Minkner, Yvonne Barth, Stefanie Bley, Angelina Ruiz-Lambides, Olaf Junge, Roger Mundry, Lars Kulik

**Affiliations:** 1 Junior Research Group of Primate Kin Selection, Department of Primatology, Max-Planck Institute for Evolutionary Anthropology, Deutscher Platz 6, 04103 Leipzig, Germany; 2 Research Group of Behavioural Ecology, Institute of Biology, University of Leipzig, Talstraße 33, 04103 Leipzig, Germany; 3 German Center for Integrative Biodiversity Research (iDiv), Deutscher Platz 5E, 04103 Leipzig, Germany; 4 Caribbean Primate Research Center, University of Puerto Rico, Medical Sciences Campus, Punta Santiago, PO Box 906, San Juan, PR 00741 USA; 5 Institute of Medical Informatics and Statistics, University Medical Center Schleswig-Holstein, Campus Kiel, Brunswiker Straße 10, 24105 Kiel, Germany; 6 Max-Planck Institute for Evolutionary Anthropology, Deutscher Platz 6, 04103 Leipzig, Germany

**Keywords:** Multi-generational pedigree, Estimates of inbreeding, Inbreeding avoidance, Early mortality, Rhesus macaques, Genetic isolation

## Abstract

**Abstract:**

When close relatives mate, offspring are expected to suffer fitness consequences due to inbreeding depression. Inbreeding has previously been quantified in two ways: using a sufficiently large panel of markers or deep and complete pedigrees over several generations. However, the application of both approaches is still limited by the challenge of compiling such data for species with long generation times, such as primates. Here, we assess inbreeding in rhesus macaques living on Cayo Santiago (Puerto Rico), a population genetically isolated since 1938, but descendant of a large set of presumably unrelated founders. Using comprehensive genetic data, we calculated inbreeding coefficients (*F*) for 2669 individuals with complete three generation pedigrees and 609 individuals with complete four generation pedigrees. We found that 0.79 and 7.39% of individuals had an *F* > 0 when using data from three and four generation pedigrees, respectively. No evidence of an increase in inbreeding over the study period (up to 23 years) was found. Furthermore, the observed mean relatedness of breeding pairs differed significantly from the distribution of parental relatedness expected as simulated based on previous reproductive data, suggesting that kin generally avoid breeding with each other. Finally, inbreeding was not a predictor of early mortality measured as survival until weaning and sexual maturation, respectively. Our results remain consistent with three estimators of inbreeding (standardized heterozygosity, internal relatedness, and homozygosity by loci) using up to 42 highly polymorphic microsatellites for the same set of individuals. Together, our results demonstrate that close inbreeding may not be prevalent *even* in populations isolated over long periods when mechanisms of inbreeding avoidance can operate.

**Significance statement:**

When close relatives mate, offspring may suffer from such inbreeding, e.g., via lower survival and/or fertility. Using (i) a large panel of genetic markers and (ii) complete three or four generation pedigrees, respectively, we show that incidences of inbreeding in a long-lived primate population are rare, even after genetic isolation for 75 years. Moreover, our simulations suggest that kin in our population generally avoid breeding with each other. Finally, the few inbred individuals detected in our large sample did not suffer from lower survival. Given that many animal species face dramatic habitat loss combined with critical population declines, our study provides important implications for conservation biology in general and for population management in particular.

**Electronic supplementary material:**

The online version of this article (doi:10.1007/s00265-016-2236-6) contains supplementary material, which is available to authorized users.

## Introduction

Individuals are considered as inbred when their parents are related to some degree. Studying inbreeding is important for our understanding of individual variation in fitness, as offspring of closely related parents are expected to have lower fitness than offspring of unrelated or distantly related parents, a phenomenon referred to as inbreeding depression (Charlesworth and Charlesworth 1987). Inbreeding increases “identity-by-descent” and therefore reduces heterozygosity of a given individual (Pemberton 2004; Slate et al. 2004; Taylor et al. 2010). Some of the deleterious effects observed in inbred offspring are lower chances of survival (Overall et al. 2005), lower fertility (Charpentier et al. 2005b), delayed development (Slate and Pemberton 2002), and a decreased immune function (Acevedo-Whitehouse et al. 2003; Rijks et al. 2008).

A wide range of inbreeding levels has been observed both in captivity and in the wild. For example, although some level of inbreeding has been reported in captive (Fredrickson and Hedrick 2002; Cassinello 2005; Ólafsdóttir and Kristjánsson 2008) and isolated populations (Marshall and Spalton 2000; Slate et al. 2000; Slate and Pemberton 2002), negligible levels were found in other populations of similar size (captive: Rzewuska et al. 2005; Dorostkar et al. 2012; Kanthaswamy et al. 2012; isolated: Overall et al. 2005). However, high to moderate levels of inbreeding have also been detected in wild populations (Liberg et al. 2005; Bensch et al. 2006; Prado-Martinez et al. 2013), with clear fitness costs at the individual level (Amos et al. 2001; Acevedo-Whitehouse et al. 2003; Bean et al. 2004; Rijks et al. 2008).

Several population characteristics are likely to lead to high levels of inbreeding over time, such as small founder population size, short-term population size reduction (bottleneck), lack of gene flow (isolation), or small population size (Marshall and Spalton 2000; Liberg et al. 2005; Bensch et al. 2006; Archie et al. 2007; reviewed in Hedrick and Kalinowski 2000; Keller and Waller 2002). Studies of inbreeding are important not only for ecology, evolution, and conservation biology, but also for population management, particularly of small, isolated, or fragmented populations being at risk of decreasing in heterozygosity (Marshall and Spalton 2000; Archie et al. 2007; for review see Hedrick and Kalinowski 2000; Keller and Waller 2002).

Two alternative approaches have been used to estimate inbreeding: (i) calculating inbreeding coefficients from pedigree data and (ii) measuring multi-locus heterozygosity from molecular markers. In the past, pedigrees were considered more accurate for estimating inbreeding (reviewed by Pemberton 2008), but even relatively few gaps influence the accuracy of estimates (Marshall et al. 2002). Particularly in wild and free-ranging populations, pedigree data tend to be incomplete and founders unknown (while assumed to be unrelated), which likely leads to inaccurate estimates of inbreeding (Keller and Waller 2002) and inbreeding depression (Taylor et al. 2015). However, all individuals of a species share common ancestry if traced back far enough in time; hence, most of their genome is identical by descent (Powell et al. 2010; Knief et al. 2015). Old coancestry is less problematic, as most recessive deleterious mutations exist at low allele frequencies and are likely to be lost over time. Large fitness effects are more likely a result of inbreeding between recent ancestors (Balloux et al. 2004; Pemberton 2004). Recent coancestry has been traditionally assessed by pedigrees, and simulation studies showed that with three generations of pedigree data, at least 80% of the variance in inbreeding is detected (Balloux et al. 2004). This is encouraging as construction of complete pedigrees with at least three generations should also be feasible for some wild or free-ranging mammalian populations.

When using genetic markers such as microsatellites (short-tandem repeats (STRs)), several studies revealed that multi-locus heterozygosity is only weakly correlated with inbreeding coefficients derived from pedigree data (e.g., Balloux et al. 2004; Slate et al. 2004; Overall et al. 2005; Taylor et al. 2010). Consequently, when compared within the same study, inbreeding depression is often detected via pedigree, but not via multi-locus markers (e.g., Slate et al. 2004; Kim et al. 2007; but see Forstmeier et al. 2012). This appears to be more problematic when using a small panel of markers (typically 5 to 15) (Balloux et al. 2004) than when marker panels are large (e.g., Hoffman et al. 2014; but see Slate et al. 2004). Nevertheless, the exact number of loci required will depend on the extent to which genome-wide heterozygosity varies among individuals (Balloux et al. 2004), which is currently unknown for most populations (Hoffman et al. 2014).

Nowadays, marker-based estimates are expected to perform better than pedigrees when a large number of markers are used. Indeed, recent simulations suggested that inbreeding can be estimated more precisely with a large number of markers than with pedigree data (Kardos et al. 2015). However, empirical comparisons using a sufficiently large panel of markers in parallel to deep and complete pedigrees are still limited (e.g., Hoffman et al. 2014; Huisman et al. 2016), although combining the methods may contribute complementary information about population viability (Bensch et al. 2006). Compiling sufficiently deep and complete pedigrees together with at least a medium number of STR markers is particularly challenging for species with long generation times, such as primates, as it requires intense sampling over decades (cf. Szulkin et al. 2007). Nevertheless, long-term studies that allow the combination of both approaches are valuable for further advances in the study of inbreeding.

The aim of the present study was therefore to investigate inbreeding in a primate population isolated for more than seven decades. To do so, we applied both genetic markers and pedigree data to estimate inbreeding in a free-ranging population of rhesus macaques (*Macaca mulatta*) on the island of Cayo Santiago (Puerto Rico, USA) that lives in several naturally formed groups. The monkeys stem exclusively from 409 wild born animals captured in 12 districts in the Lucknow area of northern India and were transferred to Cayo Santiago in 1938 (details in Carpenter and Krakower 1941; Altmann 1962; Carpenter 1972; Rawlins and Kessler 1986). Since then, no animal has been added to the island except through births. Annual reproduction among male primates is typically skewed, i.e., restricted to a few males (reviewed in Widdig 2013), which was also reported for the study population based on short-term (Widdig et al. 2004; Dubuc et al. 2011) and lifetime reproduction (Dubuc et al. 2014). Hence, a potential founder effect and the continued isolation in combination with male reproductive skew might have decreased heterozygosity and increased the chance of individuals breeding with relatives over time, leading to inbreeding depression in this population.

We had three major goals with this study. First, we aimed to evaluate the proportion of offspring produced by closely related parents and whether it has increased over time. Second, we investigated whether inbreeding is avoided in our study population by comparing the observed relatedness of actual breeding pairs with the average relatedness as expected based on a simulation considering male reproductive skew, extra-group paternities, and natal breeding as revealed by previous studies. Finally, as inbreeding has previously been shown to reduce survival (e.g., Overall et al. 2005), we tested whether inbreeding is a predictor of early mortality by investigating different life history stages. To reach our aims, inbreeding coefficients were calculated for individuals with complete pedigree data over three or four generations. Such detailed pedigree data are still extremely limited for any free-ranging primate population. To complement our pedigree data, we conducted the same analyses with three estimates of inbreeding based on up to 42 STR markers available for this population.

## Methods

### Study species

Rhesus macaques live in multi-male, multi-female groups of 8 to 180 individuals in the wild (Seth and Seth 1986). Females are philopatric and form stable matrilineal hierarchies (Gouzoules and Gouzoules 1987), while males disperse from their natal group (Lindburg 1969; Colvin 1983) between 3 to 5.5 years of age (median age = 4.5 years) (Berard 1990). Data from captivity suggest that female rhesus macaques reach sexual maturation between 2.5 and 3.5 years of age (Zehr et al. 2005) and males between 3 and 3.5 years of age (Dixson and Nevison 1997). Females usually give birth to a single offspring per season (Rawlins and Kessler 1986). Offspring can be assigned to non-overlapping birth cohorts, although cohort members may differ in age by up to 6 months. Both males and females mate with several partners during the breeding season (Hoffman et al. 2008).

### The study population and its founding

**Fig. 1:**
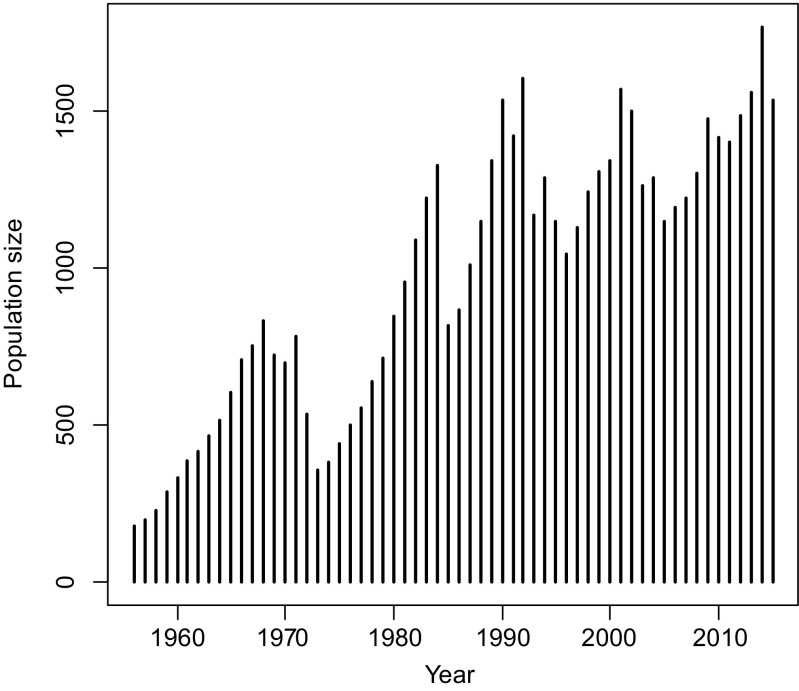
Colony growth from 1956 to 2011 showing the total number of animals recorded in the annual census

Cayo Santiago is a 15.2-ha island off the coast of Puerto Rico (18° 09′ N, 65° 44′ W) managed by the Caribbean Primate Research Center (CPRC) (Rawlins and Kessler 1986). The population stems entirely from 409 wild born animals, including 183 adult females and 40 adult males, collected from 12 districts in the Lucknow area (comprising 2500 km^2^) in the central Uttar Pradesh state of northeastern India, which were released on the island in December 1938 (Rawlins and Kessler 1986). No animals have been added since, so the population has grown exclusively through natural births. However, although a considerable proportion of infants does not survive their first year of life (14.4% of infants born between 1992 and 2014, see Supplement) while the population endures natural disasters (hurricanes and disease outbreaks), removals of animals have been necessary to control the population size (see Hernández-Pacheco et al. 2013 for details on the culling strategies and the Supplement for proportions removed). Briefly, since 1985, either entire social groups were culled or 2-year-old males and females were randomly removed to achieve an adult sex ratio of two females per male following what is observed in wild populations (Hernández-Pacheco et al. 2013). Today, the population size is maintained at around 1000 individuals living in naturally formed social groups (mean ± SD = 6.91 ± 2.94 groups during our study period) after the most intense short-term bottleneck which reduced the population to around 300 individuals by the end of 1972 (Fig. 1, for details, see Widdig et al. 2016). Animals are provisioned with a commercial monkey diet (0.23 kg/monkey/day) but spend at least 50% of their feeding time foraging extensively on natural vegetation (Marriott et al. 1989).

Data from previous studies using different methods, fewer animals, and no pedigree data suggested that the Cayo Santiago population is not inbred. A first study based on eight blood group loci sampled in 1972 reported an average heterozygosity of 0.324, indicating that a large amount of blood group variation remained (Duggleby et al. 1986). Although imprecise as a mean to measure inbreeding, a later study, assessing dyadic relatedness from multi-locus genotypes, revealed that 75% of all dyads investigated (excluding parent-offspring, full- or half-siblings) had a coefficient of relatedness of less than 0.0625 (Widdig et al. 2001). However, a more comprehensive assessment of current levels of inbreeding is now possible based on long-term genetic data (see Supplement).

Lifetime data on reproductive success suggest that males on Cayo Santiago reaching sexual maturation sire 8.7 offspring, on average (range 0–47; non-breeders 17.4% of males), while females give birth to 7.7 offspring, on average (range 0–16; non-breeders 4.5% of females) (Dubuc et al. 2014).

### Demographic information

Since 1956, trained CPRC staff has noted for all animals the ID, sex, date of birth, behavioral mother, birth group, and date of death or removal. When an individual disperses, daily census takers record its new group and check this assignment regularly for at least 2 months. If group membership remains constant, the first day seen in the new group is defined as the date of immigration for a given individual. The demographic dataset considered here comprises 11,715 animals that lived on Cayo Santiago between January 1938 and January 2011.

### Genetic data for parentage analysis

Predominantly blood samples were collected continuously from the entire population with extensive sampling efforts since 1992 in order to perform paternity analysis (see Supplement for details). Genetic sampling of individuals is routinely conducted by the CPRC during annual trapping, mainly at 1 year of age; however, for a subset of subjects born in one group (troop R) between 2005 and 2014, we maximized efforts to sample entire birth cohorts (see “Statistical analyses”). Two independent genetic databases covering different sample sets and periods as well as different sets of markers are available today. Here, we combined both genetic datasets to maximize the genetic information available (see Supplement for details).

To date, genetic information is available for 4641 animals, genotyped on an average of 27.6 ± 1.6 STR markers (mean ± SD) (see Supplement for more details). We used highly polymorphic markers showing similar characteristics with regard to the number of alleles and heterozygosity as in several wild rhesus populations (Satkoski et al. 2008; Li et al. 2013). Paternity has been determined for 3934 individuals out of the 4014 individuals (98.0%) sampled between 1992 and 2014, and maternity, as derived from behavioral observations, could be confirmed genetically for 3946 of 3996 mother-offspring pairs (98.7%). Paternity was determined using a combination of exclusion and likelihood analyses by considering all sampled potential sires present on the island around conception of the respective infant (see Supplement for details). All cases of paternity were confirmed at the 95% level (*N* = 3931) or 80% level (*N* = 3) in CERVUS 3.0 (Kalinowski et al. 2007). While it was not possible to collect genetic samples in a blind fashion, people conducting the genetic analysis were uninformed about the study question.

### Calculation of inbreeding based on pedigree

The inbreeding coefficient (*F*) of an individual was calculated in R (version 3.2.1) (R Core Team 2015) using the package “pedigree” (version 1.4) (Coster 2012). Following previous studies (e.g., Slate et al. 2004), we assumed that *F* among all founders was close to zero, which seems quite likely, given that relatedness among individuals taken from different locations spread over a large area is likely to be low or zero. The inbreeding coefficient of an offspring is equal to one half of the coefficient of relatedness (*r*) between the parents of that offspring; e.g., *F*_offspring_ *=* 0.25 corresponds to *r*_parents_ = 0.5. The use of complete and deep pedigrees (i.e., three or more generations) was shown to provide a suitable approximation of the actual *F* (Balloux et al. 2004). Therefore, we calculated *F* (i) for the subset of animals with complete four generation pedigrees (*N* = 609 individuals born in the birth seasons between 2004 and 2014) and, to increase our sample size, (ii) for animals with complete three generation pedigrees (*N* = 2669 individuals born in the birth seasons between 1992 and 2014). Both datasets included the majority of groups present during the study period and encompassed 11 and 23 study years (or birth cohorts), respectively.

### Estimating inbreeding based on genetic markers

Various marker-based estimators have been used to investigate the genome-wide diversity of populations (Pemberton 2008; Wang 2011). We calculated three commonly used estimators of inbreeding: standardized heterozygosity (SH), internal relatedness (IR), and homozygosity by loci (HL) (Slate et al. 2004; Charpentier et al. 2005b; Overall et al. 2005; Ruiz-López et al. 2009) to evaluate their performance in comparison to *F*. To achieve comparable data, the same set of animals was used as described previously. For animals with complete four generation pedigrees (*N* = 609), we had 28.4 ± 3.7 (mean ± SD) with a range of 13–41 markers available, which was similar to the subset of animals with complete three generation pedigrees (*N* = 2669) (mean ± SD = 31.0 ± 5.6, range markers = 11–42).

SH calculates the proportion of heterozygous loci divided by the mean heterozygosity of all loci typed per individual (Coltman et al. 1999). This inbreeding estimate can attain values from 0 to infinite, the lower the value, the more loci are homozygous (indicating an inbred individual). IR measures the homozygosity through counting the frequencies of shared alleles between parents giving rare shared alleles more weight than common shared alleles (Amos et al. 2001). This estimator ranges from −1 to 1, where negative values indicate offspring of more outbred parents and positive values offspring of more inbred parents. A solution for the possible underestimation of heterozygosity in animals carrying rare alleles is HL (Aparicio et al. 2006). This metric for assessing genetic variability weights heterozygosity by the diversity of each homozygous locus (Aparicio et al. 2006; Rijks et al. 2008). Loci with higher allelic variability are assumed to be more informative and thus are weighted more than others. HL ranges from 0 to 1 with 0 indicating that all loci are heterozygous and 1 suggesting that all loci are homozygous. Based on the definitions of these estimators, values of HL and SH as well as values of IR and SH were negatively correlated, while values of HL and IR were positively correlated (see Supplement). All estimators of inbreeding were calculated in R (version 2.15.1) (R Development Core Team 2010) using the package “Rhh” (Alho et al. 2010).

### Statistical analyses

#### Evaluation of inbreeding over time

We used a generalized linear mixed model (GLMM) (Baayen 2008) to assess the proportion of offspring produced by closely related parents across time in the study population. The full model comprised consecutively numbered birth cohorts (“birth season”) as a fixed effect and the IDs of the birth group, the dam, and the sire as random effects. Since it has been shown that neglecting random slopes can lead to drastically inflated type I error rates (i.e., false significance; Schielzeth and Forstmeier 2009; Barr et al. 2013), we included random slopes of birth season within sire and group but not within dam since for too many dams, we had only one birth in the data (making the random slope nearly “unidentifiable”; Barr et al. 2013). On a biological level, such random slopes account for the possibility that, for instance, the change of inbreeding over time varies among groups (a possibility that seems likely, given that groups vary considerably with regard to their size or number and size of neighboring groups) or sires (an option conceivable, too, since males might be of different attractiveness to females). Initially, we used *F* as a binary response (yes for *F* > 0, no for *F =* 0), which revealed “false convergence.” We thus transformed the *F* values into ranks, where the smallest value, *F =* 0, was set to zero, *F =* 0.0625 to 1, *F =* 0.125 to 2, and *F =* 0.25 to 3 and used a Poisson model. We fitted two such models, one based on all animals for which *F* could be calculated for three complete generations and one included all animals with four complete generations. The model was implemented in R (version 3.2.1) (R Core Team 2015) using function “glmer” of the R package “lme4” (version 1.0–8) (Bates et al. 2015). The *P* values for birth cohort were determined using a likelihood ratio test (Barr et al. 2013). Overdispersion was not an issue (three generations: *χ* ^2^ = 1.567, *df* = 2667, *P* = 1, dispersion parameter = 0.001; four generations: *χ* ^2^ = 56.867, *df* = 607, *P* = 1; dispersion parameter = 0.0934); however, there was indication of underdispersion, presumably reflecting the small number of inbreeding cases observed in our dataset.

In three additional pairs of models, we used the same approach, but *F* was replaced by one of the three inbreeding estimators SH, IR, and HL, respectively. Due to the normal distribution of the response, we here applied a Gaussian error distribution, i.e., used a linear mixed model (LMM), fitted using the function “lmer.” To assess the model stability, we compared the estimates derived by a model based on all data with those obtained from models with the levels of the random effects excluded one at a time. These revealed that the models were stable.

Finally, we calculated for each model the marginal *R* ^2^ (“variance explained”), which was suggested as an appropriate measure for the goodness of fit of a model (Nakagawa and Schielzeth 2013).

#### Evaluation of inbreeding avoidance on Cayo Santiago

To investigate whether relatives avoid breeding in our study population, we compared the observed average relatedness of breeding pairs revealed from pedigree data with the average relatedness expected based on a simulation considering previous reproductive data using a permutation test (Adams and Anthony 1996; Manly 1997). Following previous studies (e.g., Keller and Arcese 1998; Szulkin et al. 2009; Rioux-Paquette et al. 2010), we ran different simulations of the natural scenario based on published data (details in the following). To do so, we calculated relatedness from pedigrees using an R script (available upon request from the authors) for all potential breeding pairs formed by each of the available mothers of the offspring born after 1996 with a randomly chosen potential sire (chosen from all males alive and mature at the time that each of these offspring was conceived).

Several studies on Cayo Santiago revealed that male reproductive success is moderately skewed compared to other species. For example, genetic paternity success in six consecutive birth cohorts of one large group (troop R) revealed that the most successful male sired on average 24% of offspring (Widdig et al. 2004) with an average of 29.3% of group males reproducing across years (AW, unpublished data). As a high male reproductive skew could increase the likelihood of inbreeding (if age cohorts are, e.g., sired by one or two males only) and to rule out the possibility that selective culling might have reduced the likelihood of inbreeding, we used a higher skew than observed in the study population for our simulation. The same study showed that 40.9% of all infants were sired by group males of an age between 9 and 11 years (Widdig et al. 2004). Hence, we randomly chose only 20% of the males with an age between 9 and 11 years per group as potential sires to contribute to a given birth cohort per group in order to simulate male reproductive skew. Furthermore, a previous study in group R found that, on average, 24% of offspring were assigned to sires belonging to different social groups (hereafter extra-group paternities) (Widdig et al. 2004). Hence, in our simulation, 24% of offspring were randomly assigned to potential sires outside of their natal group. Finally, as 16% of offspring born in the population were sired by natal males prior to dispersal (AW, unpublished data), we consequently also included natal males as potential sires in our simulation. In summary, in each simulation, 24% of offspring were randomly assigned to extra-group males and 16% of offspring to natal males and the remaining 60% of offspring were assigned to non-natal group males of which we considered only 20% of the available group males to account for reproductive skew (i.e., simulating no natal dispersal). To investigate whether the proportion of natal males could affect the average relatedness occurrence, we ran a second simulation using the same approach as described previously, but excluding natal males as potential breeders (i.e., simulating natal dispersal of all males).

To minimize the bias due to incomplete pedigree data, we used only potential (and actual) breeding pairs with complete pedigrees for at least three generations. This simulation included 966 offspring (born after 1996), their respective mothers (*N* = 379), and all of their potential sires (*N* = 620). We repeated each simulation 1000 times to generate 1000 different datasets (into which we included the observed data as one dataset) where mothers were randomly paired with a potential sire in order to obtain the parental relatedness expected based on a simulation considering previous reproductive data. We finally determined the *P* value as the proportion of simulations revealing an average relatedness of parents at least as small as the actually observed value. If this reveals significance, it means that inbreeding was avoided.

#### Testing inbreeding as a cause of early mortality

We considered two different life history stages in this analysis (survival until 1 year of age and up to sexual maturation). First, we tested whether inbreeding was a possible cause of early infant mortality (survival until 1 year of age, i.e., weaning age). To this end, we calculated *F* for a subset of infants of our study population. Given that infant mortality in our population is highest within the first year of life (Blomquist 2013), genetic sampling at 1 year of age in general was never complete for an entire birth cohort (see Supplement). In addition, primate mothers carry dead infants for several days (e.g., Sugiyama et al. 2009), making sampling of dead infants extremely challenging under free-ranging conditions. To investigate inbreeding as a potential cause of mortality, we used data from one group (troop R) by maximizing sampling efforts for all 610 infants born between 2005 and 2014. For this subset, we either aimed at collecting the following: (i) hair samples as soon as possible after birth (1–5 months of age) during trapping, (ii) fecal samples if infants were considered sick or weak, or (iii) hair or tissue samples if infants were found dead (i.e., after the mother gave up carrying the infant). In total, we were able to sample 533 of the 610 live births (87.4%). To evaluate our sampling effort, we compared birth cohorts (or years) with a high sampling rate (>90%, mean ± SD 92.92 ± 0.03) with years of lower sampling rate (<90%, mean ± SD 77.88 ± 0.11) with regard to the mean observed inbreeding (measured as parental relatedness). Subsequently, we calculated 95% confidence intervals and found them clearly overlapping (years with sampling rates >90%, 0.000 to 0.005; years <90%, 0.002 to 0.007) indicating no obvious differences between the amounts of inbreeding detected in years of different sampling efforts.

For 516 of these infants, we were able to determine complete pedigrees for three generations, which revealed only three cases of inbreeding in this subset (0.58%). However, to reveal more distant events of inbreeding with a lower *F* value (Balloux et al. 2004), we incorporated the fourth generation by accepting incomplete pedigrees of all 516 infants, too, leading to a total of 38 inbred infants (7.36%) (*F* range 0.0313–0.25) in this subset. Of the 516 infants considered, 40 died (7.75%), while 15 were removed (2.91%) from the population before completing the first year of life. In our analysis on early infant mortality, we did not include removed subjects, leading to a total number of 501 individuals. However, this did not bias our sample as the mean *F* values were higher for infants not removed (0.004) (including dead infants) than for removed individuals (0.000).

Previous studies indicated that effects of inbreeding depression on mortality might increase until maturation (e.g., Ralls et al. 1988). Therefore, evaluating inbreeding depression based on a single (and early) life history stage might underestimate the costs of inbreeding (Szulkin et al. 2007; Grueber et al. 2010). Following a previous study (Bilski et al. 2013), in addition to survival until 1 year of age, we also looked at survival to the time of sexual maturation. Given the age of maturation reported in a previous study (see above), we assumed that by the age of 4 years both sexes have reached sexual maturation. Of the 516 individuals born, 77 died (14.92%), while 160 individuals were removed (31.01%) before completing the fourth year of life, but again, this did not create a bias of our sample as the mean *F* value was higher for individuals not removed (0.004) than for individuals who were removed (0.003). Among the removed subjects, there were a few inbred animals, which reduced the total number of inbred individuals from 38 (see above) to 27 individuals (7.58%) in this subset (*F* range 0.0313–025). Of the 516 offspring born, we did not consider the 160 subjects removed before maturation, which resulted in a total number of 356 subjects included in this analysis. Note that due to the long life span in this species, our pedigree data were not sufficient to consider the effect of inbreeding on lifetime reproductive success.

We applied a mixed Cox regression using the function “coxme” provided by the R package coxme (Therneau 2015) in order to investigate whether inbreeding caused mortality before 1 or 4 years of age, respectively. As the Cox regression requires, we used as response the number of surviving days and the appropriate status (dead/alive) at the end of the period considered (one or 4 years of age, respectively, for individuals that survived or the age at which the infant died). Hence, animals surviving at least 1 or 4 years of their lives were assigned as being 365 or 1460 days old, respectively. Per response (survival until 1 year of age or maturation), we fitted four separate Cox models including as predictor either *F* (calculated up to four generations deep) or the three estimators of inbreeding (SH, IR, HL), respectively. As random effects, we included the identity of the mother and the father (as several of them occurred repeatedly in the dataset) in all models. As our dataset included very few cases of inbreeding, we additionally used GLMMs with binomial error structure and logit function. As response, we used a variable indicating whether or not a given infant survived at least 1 or 4 years of their lives, respectively. As fixed effects and random effects, we used the same set as described previously. The models were implemented in R (version 3.2.1) (R Core Team 2015) using function glmer of the R package lme4 (version 1.0–8) (Bates et al. 2015). The *P* values for the fixed effects were determined using a likelihood ratio test (Barr et al. 2013).

## Results

### General results

Using complete pedigrees encompassing three generations, we found an *F* > 0 for 21 out of 2669 individuals investigated (0.79%) with 0.43 to 2.03% of inbred offspring born in 12 out of 23 birth cohorts considered*.* However, looking at four complete generations of pedigrees, we found 45 out of 609 individuals (7.39%) with *F* > 0 including 1.82 to 8.41% of offspring born in all 11 birth cohorts considered. As theoretically expected (Balloux et al. 2004), *F* values decreased, while the proportion of inbred offspring increased from three to four generation pedigrees (compare Tables 1 and 2).

**Table 1:** Inbreeding detected in the three-generation pedigree data

*F*	Parent generation with inbreeding noted between	Kin line	*N* cases
0.25	Daughter/sire	Paternal	2
0.125	Paternal half-sibs	Paternal	18
0.125	Maternal half-sibs	Maternal	1

Note that cases of inbreeding detected in three-generation pedigrees were only considered in the four-generation analysis if the four-generation pedigree was complete. Likewise, cases detected only in the four-generation pedigree (e.g., the common ancestor is a great grandparent) remain undetected when considering only three generations

**Table 2:** Inbreeding detected in the four-generation pedigree data

*F*	Parent generation with inbreeding noted between	Kin line	*N* cases
0.125	Paternal half-sibs	Paternal	4
0.03125	Cousins	Maternal	1
0.03125	Cousins	Paternal	4
0.03125	Cousins	Maternal and paternal	29
0.0625	Uncle/niece	Maternal and paternal	1
0.0625	Aunt/nephew	Maternal and paternal	3
0.0625	Uncle/niece	Paternal	2
0.0625	Aunt/nephew	Paternal	1

Same note as in Table 1

The 21 inbred offspring detected within the three generation pedigrees (i.e., up to the grandparent) (mean *F* = 0.14) were almost exclusively inbred within the paternal line. Specifically, we detected a total of 18 cases of inbreeding between paternal half-siblings (in 11 of these, the sire was still in his natal group and often high ranking, whereas all the remaining cases were the result of extra-group paternity), and in two cases, inbreeding concerned father-daughter pairs (with one case of an extra-group paternity). The remaining case concerned inbreeding between maternal half-siblings who were never coresident in the same group (i.e., the brother left the group before the sister was born) with the inbred offspring resulting from extra-group paternity (Table 1).

Looking at the 45 inbred offspring within four generation pedigrees (i.e., up to great grandparent, hence inbreeding deeper in the pedigree was revealed) (mean *F* = 0.04), the highest *F* was found again among paternal kin (i.e., paternal half-siblings); however, the majority of parents producing inbred offspring were related via both the maternal and the paternal lines (Table 2, see Fig. S1a–c in Supplement).

#### Evaluation of inbreeding over time

To assess inbreeding over time, we fitted a GLMM or LMM, separately for each inbreeding measure including individuals of consecutive birth cohorts. Regardless of the inbreeding measure used as response, the results revealed that the level of inbreeding did not change significantly over time with the results of the model being consistent for three and four generation pedigree data (Tables 3 and 4). Over all models, the marginal *R* ^2^ values ranged between 0.001 and 0.003, which supports our finding that variance of inbreeding was not well explained by birth season.

**Table 3:** Results of the GLMM and LMMs testing inbreeding over time assessed by *F* and inbreeding estimates (IR, SH, and HL) using the same subset of 2669 individuals (three complete generation pedigrees)

	Est	SE	*χ* ^2^	*df*	*P* values	2.5% CL	97.5% CL
*F*							
Intercept	−15.986	2.563				−21.169	−15.501
Birth season	−0.198	1.983	0.060	1	0.806	−1.151	0.220
IR							
Intercept	−0.001	0.002				−0.005	0.005
Birth season	0.001	0.002	0.062	1	0.804	−0.003	0.005
SH							
Intercept	0.001	0.003				−0.004	0.007
Birth season	−0.001	0.003	0.081	1	0.776	−0.006	0.003
HL							
Intercept	−0.001	0.002				−0.004	0.003
Birth season	0.001	0.002	0.622	1	0.430	−0.003	0.005

**Table 4:** Results of the GLMM and LMMs testing inbreeding over time assessed by *F* and inbreeding estimates (IR, SH, and HL) using the same subset of 609 individuals (four complete generation pedigrees)

	Est	SE	*χ* ^2^	*df*	*P* values	2.5% CL	97.5% CL
*F*							
Intercept	−5.762	0.496				−11.021	−0.815
Birth season	−0.180	0.363	0.325	1	0.569	−6.563	0.508
IR							
Intercept	−0.001	0.005				−0.010	0.008
Birth season	−0.005	0.005	0.907	1	0.341	−0.014	0.003
SH							
Intercept	0.000	0.006				−0.011	0.012
Birth season	0.007	0.005	1.501	1	0.221	−0.002	0.019
HL							
Intercept	0.000	0.004				−0.008	0.007
Birth season	−0.005	0.004	1.215	1	0.270	−0.013	0.003

#### Evaluation of inbreeding avoidance on Cayo Santiago

Our observed relatedness of actual breeding pairs showed that 92.4% of pairs were unrelated (Table 5). Although the distribution was skewed towards zero due to the majority of unrelated breeders, it was best represented by its mean (*r* = 0.008 ± 0.033, mean ± SD, range 0.0–0.5) in comparison to other distribution measures. Therefore, we also used the mean to compare the observed and simulated populations.

**Table 5:** Distribution of parental relatedness in the actual breeders (based on 966 offspring)

*R*	0	0.0625	0.125	0.25	0.5
*N*	893	42	25	5	1

*N* represents the number of actual breeders per degree of relatedness (*r*)

**Fig. 2:**
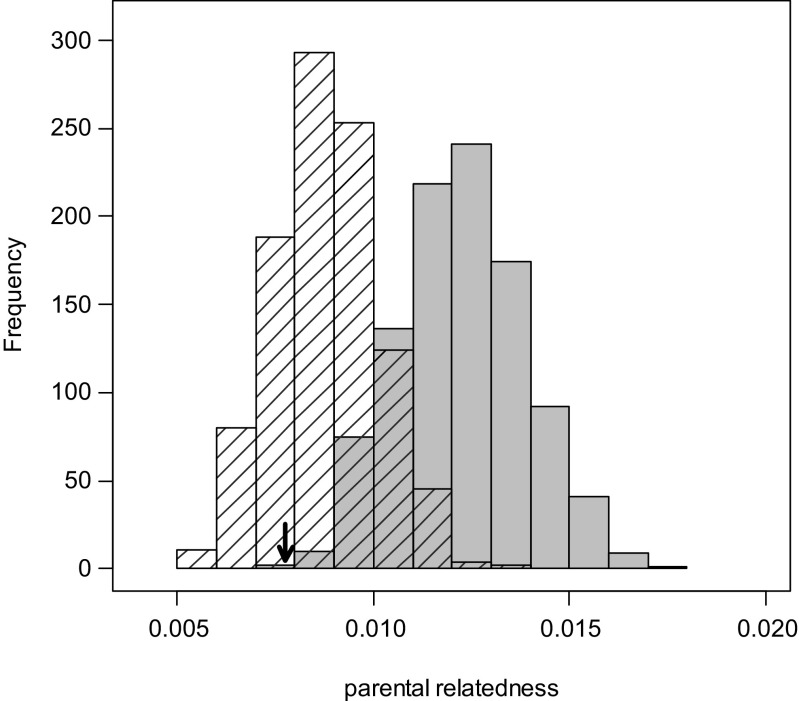
Simulated *r*-value distributions considering male reproductive skew and extra-group paternity. While the distribution depicted by *gray bars* was simulated including natal breeders, they were excluded in the second simulation (*hatched bars*). The *arrow* indicates the observed mean parental relatedness. Comparing both distributions, this illustrates that individuals seem to avoid breeding with kin which decreases the chance of inbreeding

The observed mean relatedness of pairs that reproduced in the population (arrow in Fig. 2) was clearly below the distribution of parental relatedness expected while considering reproductive skew, extra-group paternity, and natal breeders (gray bars in Fig. 2, permutation test, *P* = 0.001), suggesting inbreeding avoidance. Over this set of simulations (allowing natal breeding), the relatedness values had a mean of 0.012 ± 0.002 (mean ± SD) and ranged between 0.008 and 0.017, while the proportion of values >0 within each simulation was 0.093 ± 0.009 (mean ± SD). To further investigate the importance of natal breeders on the occurrence of inbreeding, we ran the same simulation again, this time excluding the possibility of natal breeding (i.e., simulating natal dispersal of all males). This led to smaller mean relatedness values of potential breeders (hatched bars in Fig. 2) and a non-significant difference between the observed and expected mean relatedness (permutation test, *P* = 0.204). Over this set of simulations (excluding natal breeding), the relatedness values had a mean of 0.009 ± 0.001 (mean ± SD) and ranged between 0.005 and 0.014, while the proportion of values >0 within each simulation was 0.073 ± 0.008 (mean ± SD). Comparing both distributions, this illustrates that natal breeding indeed increases the chance of inbreeding. Overall, our results reveal that kin breed less often than expected based on a simulation considering male reproductive skew, extra-group paternities, and natal breeding, suggesting avoidance of breeding with kin.

#### Testing inbreeding as a cause of early mortality

The Cox regression using *F* for three generations as a predictor did not converge, as expected, given that only two inbreeding cases were detected. Using *F* calculated for up to four, partly incomplete generations, the results of the Cox regression did not provide any evidence of inbreeding affecting the probability of early infant death (Table 6). Similarly, no effect of inbreeding was found on the probability to survive until maturation (Table 7). The results did not change when using the three other inbreeding estimates (Tables 6 and 7) or when applying binomial regression analyses on these data (Tables S6 and S7 in Supplement).

**Table 6:** Results of the Cox regressions testing the influence of *F* and inbreeding estimates (IR, SH, and HL), respectively, on survival up to 1 year of life

Survival within the first year of life				
Predictor	Estimate	SE	*Z*	*P*
*F* (4 gen)	4.954	7.065	0.700	0.480
IR	1.259	1.417	0.890	0.370
SH	−1.333	1.329	−1.000	0.320
HL	1.822	1.888	0.970	0.330

**Table 7:** Results of the Cox regressions testing the influence of *F* and inbreeding estimates (IR, SH, and HL), respectively, on survival up to sexual maturation

Predictor	Estimate	SE	*Z*	*P* values
*F* (4 gen)	2.978	5.937	0.500	0.620
IR	0.547	1.120	0.490	0.620
SH	−0.570	1.051	−0.540	0.590
HL	0.815	1.490	0.550	0.580

## Discussion

This study assessed the incidence of inbreeding based on deep and complete pedigree data in parallel to a large panel of genetic markers in a long-lived primate species. Overall, the results indicate that, even after more than seven decades of genetic isolation, events of inbreeding are rare in the rhesus macaque population of the Cayo Santiago island. First, we found no evidence of an increase in inbreeding over up to 23 years, regardless of the inbreeding measure used. Second, the observed mean relatedness of pairs that had reproduced in the population was clearly below the distribution expected based on a simulation, suggesting that breeding with kin occurred less often than expected by chance and may be avoided. Finally, when investigating whether mortality until weaning or maturation (1 or 4 years of age, respectively) was influenced by inbreeding, we found no obvious effects for either life history stage. Overall, this suggests that the low degree of inbreeding found in the study population did not result in fitness costs as measured by offspring survival.

Several mechanisms for inbreeding avoidance, such as sex-biased dispersal, extra-group paternity, and kin recognition, have been suggested and reported in a wide range of mammalian species (Kuester et al. 1994; Pusey and Wolf 1996; Goossens et al. 2001; Takahata et al. 2002; Archie et al. 2007; Costello et al. 2008). There is some evidence for all three mechanisms in the study population. First, most males on Cayo Santiago disperse from their natal group, leaving all familiar female maternal relatives behind (Berard 1990). Since male reproduction is skewed (Widdig et al. 2004; Dubuc et al. 2011, 2014), only a small proportion of males each produce several offspring who are then related as paternal half-siblings. Male natal dispersal at puberty should most likely separate paternal half-siblings of the opposite sex born in the same group before they start reproducing (Kuester et al. 1994); however, a delay in natal dispersal increases the chance of mating between paternal half-siblings of the opposite sex (Alberts 1999). As male rhesus macaques change groups several times during their lives (Berard et al. 1994) and reproduce in different groups, paternal half-siblings can also be born and grow up in different groups. This could potentially result in matings between unfamiliar paternal half-siblings of the opposite sex either via extra-group paternity or if males end up in a non-natal group containing paternal half-sisters, as found in this study. Father-daughter inbreeding is less expected given that sires tend to disperse before their daughters mature (Clutton-Brock 1988; Alberts and Altmann 1995). This seems also true for the study population given that mean male tenure is about 2 years (Manson 1995) and that we observed only two cases of inbreeding in father-daughter pairs. Hence, our results are in line with previous suggestions that even when only few other groups are available, dispersal should result in low inbreeding coefficients (Cheney and Seyfarth 1983).

Second, inbreeding avoidance might also be facilitated by the occurrence of extra-group paternities. Extra-group paternity could allow individuals to choose unrelated breeding partners, particularly if inbreeding risk is high in the natal group. Extra-group paternities indeed are regularly observed on Cayo Santiago, with 24% of offspring from six birth cohorts within one social group being sired by non-group males (Widdig et al. 2004). However, comprehensive studies investigating whether rates of extra-group paternity correlate with the risk of inbreeding in the natal group are lacking.

Finally, the recognition of related individuals and a preference for unrelated mating partners were proposed to aid in inbreeding avoidance (Widdig 2007). Evidence for kin bias (implying kin recognition) has been found for both maternal and paternal kin in the study population (Kapsalis and Berman 1996; Widdig et al. 2001). The effect of paternal kin bias was always weaker than that of maternal kin bias (reviewed in Widdig 2013), indicating that the degree of familiarity, and hence the reliability of kin recognition, is lower for paternal kin (see below). Nevertheless, there is growing evidence for kin recognition among paternal kin requiring alternative mechanisms, such as phenotype matching (Pfefferle et al. 2014a, b; Levréro et al. 2015).

Although our study lacks mating data, previous studies on mating behavior revealed that female rhesus macaques show sexual aversion to male kin and prefer to mate with unrelated males (Manson and Perry 1993). Our genetic data on reproductive output, together with our simulation, now suggest that mating between kin might actually be avoided. It is important to note that our study is unable to distinguish between pre-copulatory (avoidance of mating with relatives) and post-copulatory (e.g., failure to fertilize, spontaneous abortion due to mating with relatives) mechanisms. For example, father-daughter matings seem actively avoided in white-faced capuchin monkeys, *Cebus capucinus*, although alpha males in this species monopolize most paternities and are often still present when daughters matured (Muniz et al. 2006). Future studies should investigate the underlying mechanism in more detail and additionally explore the possibility of early fetal loss when close paternal kin breed (cf. Muniz et al. 2006).

The results of our study revealed that 0.79% of the 2669 individuals with complete three generation pedigrees had an *F* > 0, which increased to 7.39% when four complete generation pedigrees of 609 individuals were considered. As theoretically expected, the proportion of inbred individuals detected increased (Balloux et al. 2004), while the average *F* decreased with the number of generations included, because additional inbreeding events appeared further up in the pedigree. When considering three generation pedigrees, inbreeding among paternal half-siblings was predominant, while when considering four generations (and hence inbreeding events deeper in the pedigree), the parent pair producing the inbred offspring was more often related via both the maternal and the paternal lines. Inbreeding at the half-sibling level (*r* = 0.25) was more likely within the paternal line. This is likely for at least two reasons. First, there is a higher availability of paternal than maternal half-siblings in general, but in particular within the same age cohort, due to male skew in annual reproduction and females mainly giving birth to one offspring per year (Widdig et al. 2004). Hence, sons of successful sires face a high number of same-aged paternal half-sisters that they can potentially mate with, particularly when dispersal is delayed. Second, paternal half-siblings grow up with different mothers (and hence different social environments), which results in lower familiarity among them compared to maternal half-siblings sharing the same mother and social setting (Widdig 2013). Similar, in most inbreeding cases between parents related via the maternal and paternal line, females mated with an *unfamiliar* male relative, as offspring of their male kin, even of a maternal brother, are unlikely to be familiar to them.

What seems most critical when assessing inbreeding in a given population is to evaluate potential fitness consequences due to inbreeding depression (Keller and Waller 2002). For example, in a natural population of gray seals (*Halichoerus grypus*), pups with a higher IR had a significantly lower survival (Bean et al. 2004). Likewise, a study of an island population of red deer (*Cervus elaphus*) found evidence of inbreeding depression on offspring birth weight and first year survival (Walling et al. 2011; Huisman et al. 2016). Inbreeding depression was also reported in several traits among cooperatively breeding meerkats, *Suricata suricatta* (Nielsen et al. 2012). Interestingly, pup survival did not show evidence of inbreeding depression, but survival of inbred juveniles was reduced, possibly due to the increased stress associated with reaching independence (Nielsen et al. 2012), which is similar to results reported for bush dogs, *Speothos venaticus* (Bilski et al. 2013).

Both our marker-based estimates and our inbreeding coefficient based on pedigrees detected no evidence for costs of inbreeding due to death in early infancy or until maturation (1 or 4 years of age) suggesting that there may be no inbreeding depression in the sample investigated. This is interesting given that we still detected mechanisms of inbreeding avoidance. Several possibilities are likely explanations. First, there might have been inbreeding depression in the past (potentially long before the foundation of the study population) that led to selection of inbreeding avoidance mechanisms that we find today. A second possibility is that inbreeding avoidance might not have been costly to evolve in this species and therefore may have been selected for even though costs of inbreeding are rather low, too (cf. Archie et al. 2007). Alternatively, we might have been unable to detect inbreeding depression due to a low power given the restricted number of complete generations of pedigrees or markers used (cf. Overall et al. 2005). Furthermore, food provisioning in the study population might buffer effects of inbreeding depression, despite the limited access to artificial food by infants below 1 year of age. Future studies should therefore investigate more life history stages using more comprehensive genetic data under wild conditions.

Although our results indicate that colony management did not produce a bias through selective culling of inbred individuals, our genetic sample included only 75.5% of offspring born during our entire study period (see Supplement). Hence, it might be argued that our sample could lack individuals that died due to inbreeding depression before genetic sampling. We compensated this in two ways, first by enhancing sampling effort to 87.4% of offspring when investigating the impact of inbreeding on mortality and second by restricting our analysis to individuals with complete pedigrees (except in the mortality analysis, caused by too few inbred cases). In addition, mean observed inbreeding did not differ between years with high infant sampling success (>90%) and years of lower sampling success (<90%), increasing our confidence that the lack of samples did not bias our results.

Overall, mechanisms of inbreeding avoidance appear to be effectively used within the study population, which, in combination with a sufficiently large and presumably unrelated founder population, seem to reduce inbreeding as compared to estimates from biologically relevant simulations. Our simulation further supports the importance of sex-biased dispersal as a mechanism of inbreeding avoidance as it revealed a lower expected relatedness of potential parents when excluding natal breeding. The avoidance to breed with kin, in addition to male dispersal, even with the limited options of an isolated population, seems to be sufficient to maintain heterozygosity. These results are consistent with genetic data from wild rhesus macaques reporting no inbreeding, but high gene flow between groups, likely caused by male natal dispersal, relatively short non-natal group tenure, and avoidance of consanguineous matings (Melnick et al. 1984). Both approaches, pedigree data and genetic markers, revealed the same patterns with regard to inbreeding and its potential costs. Hence, the complementary use of both approaches might lead to more robust conclusions than either method alone. This calls for more long-term studies including extended genetic, mating, and fitness data from wild or free-ranging populations as they have a great potential to increase our understanding on inbreeding and its consequences across different social systems.

In contrast to our study, previous studies on other isolated populations with much smaller founder size showed a higher degree of inbreeding events (reviewed in Keller and Waller 2002). For instance, a pedigree study of an isolated population of bighorn sheep (*Ovis canadensis*) maintained at about 30 females revealed that 18.1% of the lambs had an *F* > 0 (Rioux-Paquette et al. 2011). In addition, the probability of lamb survival for inbred females was 40% lower than for non-inbred ones (Rioux-Paquette et al. 2011). The only extensive pedigree data of a primate species so far come from an isolated mandrill (*Mandrillus sphinx*) group, with five complete generations of pedigrees including 14 founders (Charpentier et al. 2006). In this population, 30% of all offspring born were inbred, with *F* ranging from 0.0625 to 0.25 (Charpentier et al. 2006). These higher levels of inbreeding were probably caused by a more pronounced male reproductive skew in mandrills (Charpentier et al. 2005a), by the lack of dispersal in this population, and potentially also by the limited number of founders. Our results might therefore be helpful to determine an appropriate size of a given founder population in order to reduce the risk of inbreeding. However, effects of inbreeding cannot be compared directly between species, as many factors such as genetic diversity, dispersal and mating patterns, kin structure, and environmental conditions influence the potential of inbreeding (Hedrick and Kalinowski 2000; Keller et al. 2002; Edmands 2007; Pemberton 2008). Consequently, it is difficult to give a general number for the minimum or optimal founder population size, although such estimates would be desired for managed or fragmented populations. Given that many animal species face dramatic habitat loss combined with critical population declines (e.g., Campbell et al. 2008) and a loss of connectivity between subpopulations, this would be particularly important for primates serving as umbrella species.

## Electronic supplementary material

ESM 1 (DOCX 90 kb).
