# Supplementary material for: Low incidence of inbreeding in a long-lived primate population isolated for 75 years
Source: Behav Ecol Sociobiol. 2016 Dec 9;71(1):18. doi: 10.1007/s00265-016-2236-6 (PMC5145906; doi:10.1007/s00265-016-2236-6)
Supplement: Supplementary file 1 — (DOCX 90 kb) [file 265_2016_2236_MOESM1_ESM.docx]

**Supplement**

**Low incidence of inbreeding in a long-lived primate population isolated for 75 years**

Published in *Behavioral Ecology and Sociobiology*

Anja Widdig^1,2,3^, Laura Muniz^1,2^, Mirjam Minkner^1,2^, Yvonne Barth^1,2^, Stefanie Bley^1,2^ , Angelina Ruiz-Lambides^1,2,4^, Olaf Junge^5^, Roger Mundry^6^, Lars Kulik^1,2^

1) Junior Research Group of Primate Kin Selection, Department of Primatology, Max-Planck Institute for Evolutionary Anthropology, Deutscher Platz 6, 04103 Leipzig, Germany

2) Research Group of Behavioural Ecology, Institute of Biology, University of Leipzig, Talstraße 33, 04103 Leipzig, Germany

3) German Center for Integrative Biodiversity Research (iDiv), Deutscher Platz 5E, 04103 Leipzig, Germany

4) Caribbean Primate Research Center, University of Puerto Rico, Medical Sciences Campus, PO Box 906, Punta Santiago, PR 00741, USA

5) Institute of Medical Informatics and Statistics, University Medical Center Schleswig-Holstein, Campus Kiel, Brunswiker Straße 10, 24105 Kiel, Germany

6) Max-Planck Institute for Evolutionary Anthropology, Deutscher Platz 6, 04103 Leipzig, Germany

* Corresponding author: Anja Widdig, Research Group of Behavioural Ecology, Institute of Biology, University of Leipzig, Talstraße 33, 04103 Leipzig, Germany, phone: +49-0341-9736707, Fax: +49-0341-9736848, Email: [anja.widdig@eva.mpg.de](mailto:anja.widdig@eva.mpg.de)

1. ***Genetic data***

Since 1992, nearly all members of the population were sampled for genetic paternity analyses when trapped as yearlings during the annual trapping season conducted by the CPRC for the purpose of individual tattooing and tetanus inoculation (introduced in 1985; details in Kessler et al. 2015). This resulted in a genetic database including animals born between 1966 and 2015. Here, we focused on infants born between 1992 and 2014 which includes 5316 live births according to the CPRC census. According to the demographic data, 4257 of these infants reached at least one year of age (80.1%), while 766 infants were recorded as passed away within the first year of their life (14.4%) (cf. Blomquist 2013) and 293 infants were culled along with their mothers during their first months of life (5.5%).

As stressed in the main text, genetic sampling at one year of age in general was never complete for an entire birth cohort. However, during each annual trapping season, infants at the age of one year were trapped to receive their individual identification code. After return to their social group, each individual will be matched to its behavioral mother, so the baby code used before will be replaced in the demographic database by the individual identification code (‘confirmed ID’). From the total of 5316 infants recorded as live births, we were able to collect DNA from 4014 infants of confirmed IDs (75.5%), but for the remaining ones we lack a genetic sample. The lacking samples included only animals of non-confirmed IDs, either removed subjects (to control for population size, see main text) or dead subjects likely including some double entries for the same infant due to the inability to correctly identify all dead infants.

*a) University of Leipzig (UL) database*

Between 1992 and 2000, blood samples were collected from all captured monkeys of the entire island during the annual trapping season. This resulted in the first genetic database of the Cayo Santiago population established through a collaboration between the CPRC and German scientists. The initial genetic database was constructed with up to 15 microsatellite markers (Nürnberg et al. 1998; Widdig et al. 2001, 2004). After 2000, this database was maintained by AW and collaborators. In 2010, the database was transferred to the genetic laboratory of the Behavioral Ecology Research Group at University Leipzig, Germany. Sampling and genotyping continued on one study group, troop R, including all offspring of consecutive birth cohorts and new potential sires of the entire island not previously genotyped (Langos et al. 2013). Starting in 2007, all group members of group V as well as infants born between 2004 and 2009 were included (Dubuc et al. 2011, 2012). In the last decade, tissue, hair and faecal samples from alive and dead subjects were also the source of genetic material.

During this time, efforts to increase the power of analyses of the marker panel involved discontinuing genotyping at problematic markers and adding new ones (Dubuc et al. 2011; Kulik et al. 2012; Albers and Widdig 2013; Langos et al. 2013) resulting in a total of 23 markers currently present in this database. Furthermore, protocols have been optimized for faecal samples over time in order to incorporate multiple tubes (Morin et al. 2001) and a multiplex approach (Henegariu et al. 1997; Bonhomme et al. 2005). In total, 2388 unique individuals were genotyped at 18.9±3.2 (mean±SD) loci, which exhibited an average of 7.2±2.5 alleles per locus, a mean observed heterozygosity of 0.732±0.065 and a mean expected heterozygosity of 0.730±0.067. In this dataset we found no significant deviation from Hardy-Weinberg equilibrium nor the presence of null alleles, and the mean polymorphism information content was 0.688±0.080 (all values calculated for the entire UL database with CERVUS 3.0) (Kalinowski et al. 2007). Details on markers and PCR conditions are presented in Table S1 (see below).

DNA was extracted from whole blood or tissue using the DNeasy^®^ Blood Tissue Kit (Qiagen), from hair using the QIAamp^®^DNA Investigator Kit (Qiagen) and from faecal samples using the QIAamp^®^ DNA Stool Mini Kit (Qiagen). PCRs were set up in 25 µl reactions using the Type-it^®^ Microsatellite PCR Kit (Qiagen) and fluorescence-labelled primers in concentrations ranging from 0.1 to 0.4 µM, as required for each multiplex PCR. Cycling conditions consisted of 1 cycle of 5 min at 95 °C, followed by 45 cycles 30 sec at 94°, 90 sec at a specific annealing temperature (Table S1), 30 sec at 72 °C and a final extension at 72 °C for 60 min. PCR products were separated and detected on the Applied Biosystems 3130xl. Primer sequences for markers in the UL database are listed in Table S2.

*b) CPRC database*

The CPRC started another genetic database in 2008 focusing on recent cohorts of the entire population, but including also animals born before 2008 if still alive or if their DNA was still available. Whole blood DNA samples were genotyped at up to 29 markers (Rogers et al. 2006) using an automated multiplex protocol. Currently, the CPRC genetic database contains 4577 unique individuals typed at 27.7±0.5 (mean±SD) loci, which exhibited an average of 8.7±3.9 alleles per locus. Excluding the sex-linked marker (DXS2506), the data showed no significant deviation from Hardy-Weinberg equilibrium and no presence of null alleles. The mean observed heterozygosity was 0.693±0.163, the mean expected heterozygosity across loci was 0.690±0.162, and the mean polymorphism information content was 0.652±0.164 (all values calculated for the entire CPRC database with CERVUS 3.0) (Kalinowski et al. 2007). Details on marker and PCR conditions are presented in Table S3.

For this database, DNA was extracted from whole blood using an alkaline lysis method (Andrade et al. 2004). PCRs were set up in 25 µl reactions containing 30-60 ng of samples, 2.5 mM MgCl2, 200 µM dNTPs, 1X PCR buffer (Denville Scientific Inc., Metuchen, NJ), 0.7 U *Choice* Taq (Denville Scientific Inc., Metuchen, NJ) and fluorescence-labelled primers in concentrations and sequences detailed elsewhere (see supplement in Miller et al. 2010). Cycling conditions consisted of 4 cycles of 1 min at 94 °C, 30 sec at the general annealing temperature of 58 °C, 30 sec at 72 °C, followed by 25 cycles of 45 sec at 94 °C, 30 sec at 58 °C, 30 sec at 72 °C and a final extension at 72 °C for 30 min. PCR products were separated by capillary electrophoresis on ABI 3730 DNA Analyzer (Applied Biosystems, Foster City).

*c) Accordance of genotypes in both database (error rate)*

In total, 2319 individuals were typed in both datasets with an overlap of 4.17±1.94 markers (mean±SD) between databases. Accordingly, a total of 26,826 alleles were produced in both databases with 26,624 alleles being consistent across datasets (99.25%) indicating a genotype error rate of 0.008.

*d) Paternity determination for pedigree reconstruction (assignment rules and power)*

Parentage information was determined independently for each dataset and results were later compared and combined. Initially, maternity information from census records was confirmed using genotypic data available within each dataset, and this information was subsequently used in paternity analyses. Given that many offspring were assigned to sires belonging to different social groups (extra-group paternities) (Widdig et al. 2004), all mature males on the island were considered as potential sires if they fulfilled two conservative criteria: i) males had to be alive at least one month before conception of a given infant counting back 200 days from its date of birth, following a gestation length estimate of 166.5±7.4 (Silk et al. 1993), and ii) males had to be older than 1250 days of age on the birth date of the respective infant, based on the youngest age at reproduction (Bercovitch et al. 2003). This resulted in a different set of potential sires per offspring. According to the demographic database, a total of 407 (15.15%) out of all 2685 males identified as potential sires in this study (considering infants born between 1992 and 2014) could not be genotyped due to a lack of samples, however, all of these males were born prior to 1992 (the start of systematic sampling). For 98% of infants sampled between 1992 and 2014, we could assign paternity with high confidence (see below), suggesting that the lack of potential sires did not affect the success of our paternity analysis.

For the Leipzig dataset, paternity was determined using a combination of exclusions due to mismatches and likelihood calculations. First, paternity was assigned to the male having no mismatches with a given offspring across all common loci when all other males mismatched the offspring at two or more loci (strict rule). We confirmed all paternities derived from exclusion via the strict rule additionally at the 95% confidence level by the maximum likelihood method implemented in CERVUS 3.0 (Kalinowski et al. 2007), except for three cases reaching the 80% confidence level only. Second, paternity was assigned to the male having no mismatches with a given offspring across all common loci when one or more males mismatched the offspring at one locus only (relaxed rule). In these cases, the assigned sire additionally *needed* to be supported at the 95% confidence level in CERVUS 3.0 (Kalinowski et al. 2007) which was achieved in all cases. The minimum number of common loci for a paternity assignment to be valid was 12 for mother-sire-offspring trios or 15 for sire-offspring dyads.

Within the CPRC dataset, paternity was assigned using the same criteria as above. Based on a greater exclusion power due to the number of markers available in this dataset, we also assigned 153 paternities (3.8%) in which the best male had one mismatch with the offspring. This decision was justified because of the evidence of mutations based on the comparison of genotypes of three or more related individuals. All paternities assigned were supported at the 95% confidence level in CERVUS 3.0 (Kalinowski et al. 2007).

*e) Combination of parentage information from both databases*

Finally we combined both independent genetic datasets available for the Cayo Santiago rhesus population. Initially, the correspondence of IDs between datasets was established by examining parent-offspring relationships in both databases. To do so, we checked the correspondence of mother-offspring pairs and/or mother-sire-offspring trios for errors. After subsequent re-amplification of inconsistencies, two non-congruent cases were left, which were excluded from the analysis. A Masterfile was then constructed by comparing the parentage assignments for all animals present in both datasets. All congruent parentage assignments were accepted (N=1839 individuals) indicating an error rate in assignments between the two datasets of 0.001. Additionally, 48 and 2322 individuals with parentage exclusively solved in the UL or the CPRC database, respectively, were included. In this study, we used the Masterfile version from 2015-07-02, including 4641 animals, genotyped for an average of 27.6±1.6 microsatellite markers.

*f) Final paternity data used*

Paternity was determined for 3934 individuals out of 4014 individuals sampled between 1992 and 2014 (98.0%) and maternity, as derived from behavioral observations, could be confirmed genetically for 3946 of 3996 mother-offspring pairs (98.7%). Paternity was determined using a combination of exclusion and likelihood analyses as specified above. All cases of paternity were confirmed at the 95% level (N=3931) or 80% level (N=3) in CERVUS 3.0 (Kalinowski et al. 2007) using the following parameters: simulated offspring: 10,000, number of candidate fathers: 203, proportion of candidate fathers sampled: 0.61 (UL) or 0.65 (CPRC), proportion of loci typed: 0.74 (UL) or 0.95 (CPRC), proportion loci mistyped: 0.01, minimum number of typed loci: 14.

Table S1: Summary of markers in the UL database

| Locus | Number alleles | Observed Heterozygosity | Expected Heterozygosity | Polymorphism information content | Annealing Temperature (T_A_) |
| --- | --- | --- | --- | --- | --- |
| D2S367 | 4 | 0.680 | 0.664 | 0.597 | 58°C |
| D8S601 | 7 | 0.755 | 0.753 | 0.717 | 53°C |
| D20S206 | 6 | 0.746 | 0.754 | 0.717 | 53°C |
| D6S493 | 11 | 0.734 | 0.735 | 0.690 | 53°C |
| D6S474 | 7 | 0.673 | 0.661 | 0.619 | 53°C |
| D20S476 | 5 | 0.676 | 0.664 | 0.603 | 56°C |
| D12S66 | 5 | 0.734 | 0.746 | 0.702 | 51°C |
| D12S67 | 15 | 0.848 | 0.845 | 0.826 | 53°C |
| D14S255 | 6 | 0.691 | 0.694 | 0.634 | 53°C |
| D5S820 | 8 | 0.759 | 0.755 | 0.720 | 58°C |
| D5S1470 | 7 | 0.763 | 0.763 | 0.730 | 58°C |
| SCA1REP | 5 | 0.662 | 0.663 | 0.619 | 58°C |
| D6S266 | 8 | 0.844 | 0.854 | 0.836 | 58°C |
| D8S271 | 7 | 0.731 | 0.709 | 0.666 | 53°C |
| D3S1768 | 10 | 0.764 | 0.769 | 0.738 | 53°C |
| D6S1036 | 5 | 0.732 | 0.679 | 0.613 | 57°C |
| D11S2002 | 8 | 0.738 | 0.729 | 0.681 | 53°C |
| D1S548 | 5 | 0.643 | 0.639 | 0.581 | 53°C |
| D13S765 | 6 | 0.698 | 0.736 | 0.690 | 58°C |
| D15S823 | 10 | 0.840 | 0.831 | 0.812 | 58°C |
| D16S403 | 6 | 0.720 | 0.710 | 0.670 | 58°C |
| D2S1333 | 9 | 0.818 | 0.827 | 0.804 | 58°C |
| D5S1457 | 6 | 0.591 | 0.609 | 0.555 | 58°C |

Table S2: Primer sequence of markers in the UL database

| Locus | Primer Forward | Primer Reverse |
| --- | --- | --- |
| D2S367 | TTCTTTGGTCTAAGGGTCAC | AGCTTCTTGTTCACAGGTGT |
| D8S601 | TTGGCAATCACATTTCAGC | GCACAGTTGGATCTTGTGTC |
| D20S206 | TCCATTATTCCCCTCAAACA | GGTTTGCCATTCAGTTGAGA |
| D6S493 | ATCCCAACTCTTAAATGGGC | TTCCATGGCAGAAATTGTTT |
| D6S474 | TGTACAAAAGCCTATTTAGTCAGG | TCATGTGAGCCAATTCCTCT |
| D20S476 | GGTGGTGATGGAGTCTGAAG | TATTTTCTATCCTTCAAGCTACCC |
| D12S66 | TCATTTAAGCATTTGAGGGAA | AGACTTCAAAACAGACACTT |
| D12S67 | GCAACAGTTTATGCTAAAAGC | GCCTATGCAGTTCAAATCTA |
| D14S255 | AGCTTCCAATACCTCACCAA | ATCCTCTGGCTGGATAAGTG |
| D5S820 | ATTGCATGGCAACTCTTCTC | GTTCTTCAGGGAAACAGAACC |
| D5S1470 | CATGCACAGTGTGTTTACTGG | TAGGATTTTACTATATTCCCCAGG |
| Sca1Rep | AACTGGAAATGTGGACGTAC | CAACATGGGCAGTCTGAG |
| D6S266 | GTAATTAGTAGGAAGGGACCTGA | GCTGTAAGTCAACAGGGCTA |
| D8S271 | AGATGACCTGGATGAGAGTG | AACAAACTTGCTTATGAGTGTTACT |
| D3S1768 | GGTTGCTGCCAAAGATTAGA | CACTGTGATTTGCTGTTGGA |
| D6S1036 | CTTCATTCAAAGAGATAAATGGC | GGATGGAATTATTCATGGCA |
| D11S2002 | CATGGCCCTTCTTTTCATAG | AATGAGGTCTTACTTTGTTGCC |
| D1S548 | GAACTCATTGGCAAAAGGAA | GCCTCTTTGTTGCAGTGATT |
| D13S765 | TGTAACTTACTTCAAATGGCTCA | GTTCTTTTGAAACTTACAGACAGCTTGC |
| D15S823 | TTCCTCATGAGTGGCTAGGG | GTTCTTCATCTGCAAAATGGGAATGA |
| D16S403 | GTTTTCTCCCTGGGACATTT | TATTCATTTGTGTGGGCATG |
| D2S1333 | CTTTGTCTCCCCAGTTGCTA | GTTCTTTCTGTCATAAACCGTCTGCA |
| D5S1457 | TAGGTTCTGGGCATGTCTGT | GTTCTTTGCTTGGCACACTTCAGG |

Table S3: Summary of markers in the CPRC database

| Locus | Number alleles | Observed Heterozygosity | Expected Heterozygosity | Polymorphism information content | Annealing Temperature (T_A)_ |
| --- | --- | --- | --- | --- | --- |
| D10S1412 | 4 | 0.708 | 0.708 | 0.655 | 58°C |
| D11S2002 | 8 | 0.731 | 0.728 | 0.679 | 58°C |
| D11S925 | 8 | 0.706 | 0.703 | 0.652 | 58°C |
| D12S364 | 9 | 0.573 | 0.575 | 0.535 | 58°C |
| D12S67 | 19 | 0.837 | 0.841 | 0.822 | 58°C |
| D13S765 | 7 | 0.717 | 0.734 | 0.688 | 58°C |
| D15S823 | 11 | 0.838 | 0.828 | 0.809 | 58°C |
| D16S403 | 11 | 0.732 | 0.721 | 0.682 | 58°C |
| D17S1300 | 18 | 0.840 | 0.830 | 0.809 | 58°C |
| D18S537 | 3 | 0.618 | 0.608 | 0.540 | 58°C |
| D18S72 | 6 | 0.501 | 0.509 | 0.436 | 58°C |
| D1S548 | 5 | 0.653 | 0.645 | 0.587 | 58°C |
| D22S685 | 11 | 0.790 | 0.798 | 0.769 | 58°C |
| D2S1333 | 9 | 0.826 | 0.825 | 0.802 | 58°C |
| D3S1768 | 10 | 0.781 | 0.778 | 0.748 | 58°C |
| D4S2365 | 6 | 0.665 | 0.661 | 0.599 | 58°C |
| D4S413 | 8 | 0.757 | 0.753 | 0.718 | 58°C |
| D5S1457 | 7 | 0.612 | 0.621 | 0.563 | 58°C |
| D6S1691 | 8 | 0.718 | 0.704 | 0.665 | 58°C |
| D6S276 | 9 | 0.834 | 0.830 | 0.807 | 58°C |
| D6S291 | 6 | 0.562 | 0.558 | 0.528 | 58°C |
| D6S501 | 8 | 0.783 | 0.779 | 0.745 | 58°C |
| D7S513 | 15 | 0.878 | 0.870 | 0.857 | 58°C |
| D7S794 | 6 | 0.686 | 0.676 | 0.614 | 58°C |
| D8S1106 | 11 | 0.812 | 0.806 | 0.779 | 58°C |
| D9S921 | 7 | 0.563 | 0.553 | 0.513 | 58°C |
| DXS2506 | 1 | 0.000 | 0.000 | 0.000 | 58°C |
| MFGT21 | 11 | 0.751 | 0.752 | 0.718 | 58°C |
| MFGT22 | 11 | 0.616 | 0.616 | 0.584 | 58°C |

1. ***Correlations between inbreeding estimates and inbreeding coefficient***

The inbreeding estimates (SH, IR and HL) significantly correlated with each other (with high rho values), when testing individuals with pedigrees of three and four generations respectively (see Table S4 and S5).

Table S4: Spearman’s rank correlation coefficients (rho) between the inbreeding estimates (IR, SH and HL) using the animal subset with three complete generations of pedigrees

|  | N=2669 and 3 generation pedigrees | | | |
| --- | --- | --- | --- | --- |
|  | SH |  | HL |  |
|  | rho | P | rho | P |
| IR | -0.975 | **<0.001** | 0.974 | **<0.001** |
| SH |  |  | -0.989 | **<0.001** |

Table S5: Spearman’s rank correlation coefficients (rho) between the inbreeding estimates (IR, SH and HL) using the animal subset with four complete generations of pedigrees

|  | N=609 and 4 generation pedigrees | | | |
| --- | --- | --- | --- | --- |
|  | SH |  | HL |  |
|  | rho | P | rho | P |
| IR | -0.975 | **<0.001** | 0.974 | **<0.001** |
| SH |  |  | -0.990 | **<0.001** |

1. ***Binomial regression analyses for testing the influence of inbreeding on survival***

As explained in the methods, our data set included very few cases of inbreeding, therefore we additionally used GLMMs with binomial error structure and logit function which revealed also no influence of inbreeding on survival for both at least one or four year/s of their lives respectively (Table S6 and 7).

Table S6: Results of the GLMMs testing the influence of *F* and inbreeding estimates (IR, SH and HL), respectively, on survival for at least one year of life

|  | Est | SE | χ2 | df | P |
| --- | --- | --- | --- | --- | --- |
| Intercept | -3.253 | 0.274 |  |  |  |
| ***F*** | 4.726 | 10.074 | 0.236 | 1 | 0.627 |
| Intercept | -3.241 | 0.269 |  |  |  |
| **IR** | 1.376 | 1.779 | 0.624 | 1 | 0.430 |
| Intercept | -1.855 | 1.675 |  |  |  |
| **SH** | -1.385 | 1.680 | 0.709 | 1 | 0.400 |
| Intercept | -3.758 | 0.722 |  |  |  |
| **HL** | 1.914 | 2.387 | 0.672 | 1 | 0.413 |

Table S7: Results of the GLMMs testing the influence of *F* and inbreeding estimates (IR, SH and HL), respectively, on survival for at least four years of life (sexual maturation)

|  | Est | SE | χ2 | df | P |
| --- | --- | --- | --- | --- | --- |
| Intercept | -3.027 | 0.313 |  |  |  |
| ***F*** | 2.515 | 12.159 | 0.051 | 1 | 0.822 |
| Intercept | -3.017 | 0.308 |  |  |  |
| **IR** | 0.863 | 1.961 | 0.200 | 1 | 0.654 |
| Intercept | -2.038 | 1.859 |  |  |  |
| **SH** | -0.968 | 1.851 | 0.285 | 1 | 0.593 |
| Intercept | -3.355 | 0.793 |  |  |  |
| **HL** | 1.281 | 2.644 | 0.245 | 1 | 0.620 |

***4. Examples of inbreeding via the maternal and/or paternal kin line***

Figure S1a-c: Example pedigrees of inbred individuals. Males are shown as squares, females as circles and inbred offspring in black. Inbreeding via the maternal line: here the parent pair (dark grey) are maternal cousins and they share only one common female ancestor (light grey) (Fig. S1a). Inbreeding via the paternal line: here the parent pair are paternal cousins and they share only one common male ancestor (light grey) (Fig. S1b). Inbreeding via both the maternal and paternal line: here the parent pair are maternal/paternal cousins and they share one common female or male ancestor. In our example, the mother of one parent and the father of the other parent are paternal half-siblings, i.e., both parents share the same grandfather (light grey) (Fig S1c). The difference with regard to the parents in Fig. 2a and b is that from the perspective of the mother, the parents are related via the maternal line and from the perspective of the father, via the paternal line (cf. Albers and Widdig 2013).

Fig. 2b

Fig. 2c

**References:**

Albers M, Widdig A. 2013. The influence of kinship on familiar natal migrant rhesus macaques (*Macaca mulatta*). Int J Primatol. 34:99–114.

Andrade MCR, Penedo MCT, Ward T, Silva VF, Bertolini LR, Roberts JA, Leite JPG, Cabello PH. 2004. Determination of genetic status in a closed colony of rhesus monkeys (*Macaca mulatta*). Primates. 45:183–186.

Bercovitch FB, Widdig A, Trefilov A, Kessler MJ, Berard JD, Schmidtke J, Nurnberg P, Krawczak M. 2003. A longitudinal study of age-specific reproductive output and body condition among male rhesus macaques, *Macaca mulatta*. Naturwissenschaften. 90:309–312.

Blomquist GE. 2013. Maternal effects on offspring mortality in rhesus macaques (*Macaca mulatta*). Am J Primatol. 75:238–251.

Bonhomme M, Blancher A, Crouau-Roy B. 2005. Multiplexed microsatellites for rapid identification and characterization of individuals and populations of cercopithecidae. Am J Primatol. 67:385–391.

Dubuc C, Muniz L, Heistermann M, Engelhardt A, Widdig A. 2011. Testing the Priority-of-Access model in a seasonally breeding primate species. Behav Ecol Sociobiol. 65:1615–1627.

Dubuc C, Muniz L, Heistermann M, Widdig A, Engelhardt A. 2012. Do males time their mate-guarding effort with the fertile phase in order to secure fertilisation in Cayo Santiago rhesus macaques? Horm Behav. 61:696–705.

Henegariu O, Heerema N, Dlouhy S, Vance G, Vogt P. 1997. Multiplex PCR: critical parameters and step-by-step protocol. BioTechniques. 23:504–511.

Kalinowski ST, Taper ML, Marshall TC. 2007. Revising how the computer program CERVUS accommodates genotyping error increases success in paternity assignment. Mol Ecol. 16:1099–1106.

Kessler MJ, Hernández Pacheco R, Rawlins RG, Ruiz-Lambides A, Delgado DL, Sabat AM. 2015. Long-term effects of tetanus toxoid inoculation on the demography and life expectancy of the Cayo Santiago rhesus macaques. Am J Primatol. 77:211–221.

Kulik L, Muniz L, Mundry R, Widdig A. 2012. Patterns of interventions and the effect of coalitions and sociality on male fitness. Mol Ecol. 21:699–714.

Langos D, Kulik L, Mundry R, Widdig A. 2013. The impact of paternity on male–infant association in a primate with low paternity certainty. Mol Ecol. 22:3638–3651.

Miller WP, Srinivasan S, Panoskaltsis-Mortari A, Singh K, Sen S, Hamby K, Deane T, Stempora L, Beus J, Turner A, Wheeler C, Anderson DC, Sharma P, Garcia A, Strobert E, Elder E, Crocker I, Crenshaw T, Penedo MCT, Ward T, Song M, Horan J, Larsen CP, Blazar BR, Kean LS. 2010. GVHD after haploidentical transplantation: a novel, MHC-defined rhesus macaque model identifies CD28- CD8+ T cells as a reservoir of breakthrough T-cell proliferation during costimulation blockade and sirolimus-based immunosuppression. Blood. 116:5403–5418.

Morin PA, Chambers KE, Boesch C, Vigilant L. 2001. Quantitative polymerase chain reaction analysis of DNA from noninvasive samples for accurate microsatellite genotyping of wild chimpanzees (*Pan troglodytes verus*). Mol Ecol. 10:1835–1844.

Nürnberg P, Sauermann U, Kayser M, Lanfer C, Manz E, Widdig A, Berard J, Bercovitch FB, Kessler M, Schmidtke J, Krawczak M. 1998. Paternity assessment in rhesus macaques (*Macaca mulatta*): Multilocus DNA fingerprinting and PCR marker typing. Am J Primatol. 44:1–18.

Rogers J, Garcia R, Shelledy W, Kaplan J, Arya A, Johnson Z, Bergstrom M, Novakowski L, Nair P, Vinson A, Newman D, Heckman G, Cameron J. 2006. An initial genetic linkage map of the rhesus macaque (*Macaca mulatta*) genome using human microsatellite loci. Genomics. 87:30–38.

Silk JB, Short J, Roberts J, Kusnitz J. 1993. Gestation length in rhesus macaques (*Macaca mulatta*). Int J Primatol. 14:95–104.

Widdig A, Bercovitch FB, Streich WJ, Nürnberg P, Krawczak M. 2004. A longitudinal analysis of reproductive skew in male rhesus macaques. Proc R Soc B Biol Sci. 271:819–826.

Widdig A, Nürnberg P, Krawczak M, Streich WJ, Bercovitch FB. 2001. Paternal relatedness and age proximity regulate social relationships among adult female rhesus macaques. Proc Natl Acad Sci. 98:13769–13773.
